# Asymmetric, multifocal musculoskeletal pain preceding the onset of progressive supranuclear palsy: A case report

**DOI:** 10.1111/cns.13527

**Published:** 2020-12-04

**Authors:** Yi Fang, Chong‐Yao Jin, Ran Zheng, Ji‐Min Wu, Bao‐Rong Zhang, Jia‐Li Pu

**Affiliations:** ^1^ Department of Neurology Second Affiliated Hospital College of Medicine Zhejiang University Hangzhou China

**Keywords:** gait disorder, pain, progressive supranuclear palsy

Dear editors,

Pain is often reported by patients with Parkinson's disease, with a prevalence of 89% according to one study.^1^ However, pain in patients with atypical parkinsonian disorders, such as progressive supranuclear palsy (PSP) was not extensively studied and had a substantially lower prevalence according to limited available reports.^2^ Here, we present a case of a patient with asymmetric and progressive musculoskeletal pain preceding the onset of PSP with progressive gait freezing.

A 67‐year‐old female presented to our neurologic clinic complaining that she has been experiencing asymmetric pain in her lower extremities for 5 years, walking difficulty for 1 year. Five years ago, she started to experience intermittent, moderate pain in her right thigh. She did not seek for medical care, thus a detailed medical history within this period was not available. She recalled that the pain had no clear trigger, was exacerbated by motion, and spontaneously resolved after 3‐5 minutes. Her right knee, ankle, and toes were gradually involved. One year ago, her left lower extremity was involved. That is when she started to experience episodic sudden inability to produce forward steps, which was provoked by gait initiation, turns, or crossing doorways. Neither pain nor freezing of gait (FOG) was relieved after levodopa and pramipexole treatment. One month before this clinical encounter, she tried to stop dopaminergic medications, as she considered them to be not helpful. Yet she experienced worsened FOG after being noncompliant, came to our clinic for further evaluation.

On physical examination, she was noted to have FOG, hypophonia, increased muscle tone on neck and positive retropulsion test. No resting or action tremor was noted. Muscle tone and strength on extremities were normal. Ocular movements, cerebellar functions were intact. The patient reported no subjective cognitive decline and mood disturbance, with a Mini‐Mental State Exam score at 25/30, Hamilton Anxiety Rating Scale score at 5/56, and Hamilton Depression Rating Scale score at 5/56. No overt structural abnormality was found on MR scan, making vascular parkinsonism, and normal pressure hydrocephalus unlikely (Figure 1). Midbrain to pons ratio was 0.50 on midline sagittal MRI, which was suggestive of PSP.^3^ Dopamine transporter single‐photon emission computerized tomography scan revealed bilateral diffuse striatum dopaminergic deficit, more prominent on left head of caudate (Figure 1), which was commonly seen in PSP.^4^ However, a tau PET scan was not available in our institution.

**FIGURE 1:**
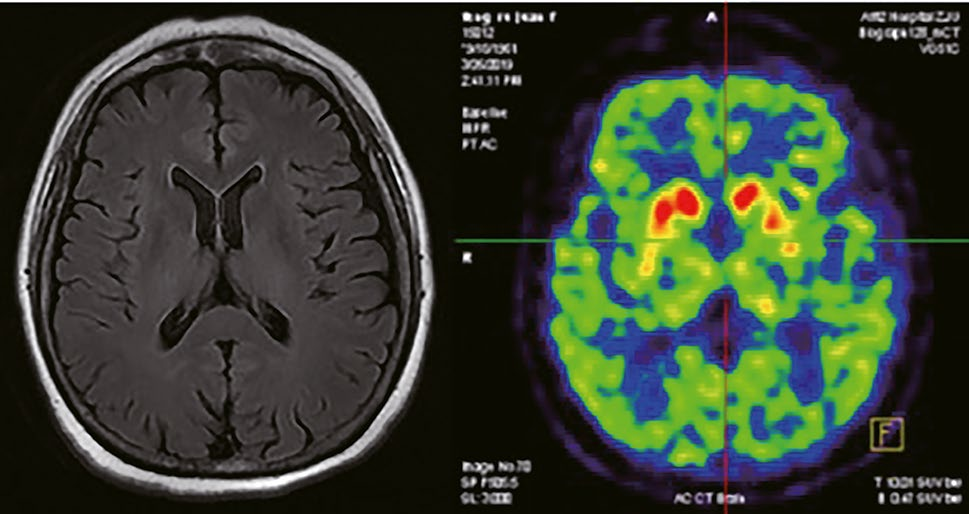
No dialted ventricles or white matter lesions found on MR axial T2‐weighted‐Fluid‐Attenuated Inversion Recovery (T2‐FLAIR). DAT revealed bilateral striatum dopaminergic deficit, more prominent reduction observed in left body of caudate

A clinical diagnosis of PSP with progressive gait freezing was made according to the movement disorder society PSP diagnostic criteria.^5^ It is likely that this patient had a rare subtype, Pure akinesia with gait freezing (PAGF), according to the diagnostic criteria provided by Williams and colleagues: gradual onset of freezing of gait or speech; absence of limb rigidity and tremor; no dementia or ophthalmoplegia in the first 5 years; no sustained response to dopaminergic medications; and no imaging changes suggestive of a vascular etiology.^6^ Although patients with PSP are poorly responsive to levodopa, therapies with Coenzyme Q10, rasagiline, devunetide, anti‐tau medications are likely beneficial.^7^

Interestingly, this patient had asymmetric progressive multifocal joint pain preceding the onset of gait freezing. Antinuclear antibody, anti‐neutrophil cytoplasmic antibodies, rheumatoid factor, cyclic citrullinated peptides, erythrocyte sedimentation rate are within normal limit. There were no signs of joint deformity, rash, swelling, increased temperature on skin. Besides, this patient did not have mood disturbances at the onset of pain. Considering the fact that pain in right lower extremity was involved first and more severe, and the fact that her dopaminergic deficit was more prominent on left on DAT scan, we speculated her pain was potentially related to neuropathologic changes that contributes to FOG.

Huge heterogeneity exists in terms of painful sensations in association with PD. It could be musculoskeletal, neuropathological, visceral, cutaneous, and radicular. Pain in PSP patients, on the other hand, has not been extensively studied. Common causes of musculoskeletal pain in idiopathic PD, such as rigidity, akinesia, and dystonia, could not account for pain in this patient. We suspect that the asymmetric progressive pain possibly originates from neuropathologic changes underlying PAGF. Dopaminergic deficits in basal ganglia causing “central pain” is a potential explanation.^8^ In addition, degenerative changes accompanying the natural course of PSP, such as in descending inhibitory fibers within brainstem, likely contribute to altered pain threshold in PSP.^9^ Locus ceruleus, previously found to be atrophied in patient with PAGF,^10^ was well established to be involved in chronic pain.^11^ Pain in this patient moderately relieved after administration of pregabalin, while duloxetine or gabapentin did not achieve satisfying effect. Nonpharmalogical approach, such as high‐frequency spinal cord stimulation,^12^ was recommended for this patient.

To summarize, we present here a patient who had musculoskeletal pain preceding the onset of PSP with progressive gait freezing. We propose that degenerative changes of PSP contribute to this unusual presentation of prodromal pain. Pain in atypical parkinsonism has not been extensively reported and studied, further investigations are warranted.

## CONFLICT OF INTEREST

The authors declare no conflict of interest.

## Funding information

This work was supported by the National Natural Science Foundation of China [grant No. 81771216 and 81520108010].

## ETHICS APPROVAL

The study was approved by the ethics review boards of the Second Affiliated Hospital of Zhejiang University.

## CONSENT TO PARTICIPATE

The patient provided written consent for participation.

## CONSENT FOR PUBLICATION

The patient provided written consent for disclosure of medical information and images.
